# Gastric microbiome changes in relation with *Helicobacter pylori* resistance

**DOI:** 10.1371/journal.pone.0284958

**Published:** 2023-05-18

**Authors:** Astri Dewayani, Kartika Afrida Fauzia, Ricky Indra Alfaray, Langgeng Agung Waskito, Dalla Doohan, Purwo Sri Rejeki, Mohammed Abdullah Alshawsh, Yudith Annisa Ayu Rezkitha, Yoshio Yamaoka, Muhammad Miftahussurur

**Affiliations:** 1 Oita University Faculty of Medicine, Department of Infectious Disease Control, Yufu, Oita, Japan; 2 Faculty of Medicine, Department of Anatomy, Histology and Pharmacology, Universitas Airlangga, Surabaya, Indonesia; 3 Institute of Tropical Disease, *Helicobacter pylori* and Microbiota Study Group, Universitas Airlangga, Surabaya, Indonesia; 4 Oita University Faculty of Medicine, Department of Environmental and Preventive Medicine, Yufu, Oita, Japan; 5 Faculty of Medicine, Department of Public Health and Preventive Medicine, Universitas Airlangga, Surabaya, Indonesia; 6 Faculty of Medicine, Department of Medical Physiology and Biochemistry, Universitas Airlangga, Surabaya, Indonesia; 7 Faculty of Medicine, Department of Pharmacology, Universiti Malaya, Kuala Lumpur, Malaysia; 8 Faculty of Medicine, School of Clinical Sciences, Nursing and Health Sciences, Monash University, Clayton, VIC, Australia; 9 Faculty of Medicine, Department of Internal Medicine, University of Muhammadiyah Surabaya, Surabaya, Indonesia; 10 Department of Medicine, Gastroenterology and Hepatology Section, Baylor College of Medicine, Houston, Texas, United States of America; 11 Research Center for Global and Local Infectious Diseases, Oita University, Yufu, Oita, Japan; 12 Faculty of Medicine, Department of Internal Medicine, Division of Gastroentero-Hepatology, Dr. Soetomo Teaching Hospital, Universitas Airlangga, Surabaya, Indonesia; UAE University: United Arab Emirates University, UNITED ARAB EMIRATES

## Abstract

**Introduction:**

Inadequate antimicrobial treatment has led to multidrug-resistant (MDR) bacteria, including *Helicobacter pylori* (*H*. *pylori*), which one of the notable pathogens in the stomach. Antibiotic-induced changes in the microbiota can negatively affect the host. This study aimed to determine the influence of *H*. *pylori* resistance on the diversity and abundance of the stomach microbiome.

**Methods:**

Bacterial DNA was extracted from biopsy samples of patients presenting dyspepsia symptoms with *H*. *pylori* positive from cultures and histology. DNA was amplified from the V3-V4 regions of the 16S rRNA gene. In-vitro E-test was used to detect antibiotic resistance. Microbiome community analysis was conducted through α-diversity, β-diversity, and relative abundance.

**Results:**

Sixty-nine *H*. *pylori* positive samples were eligible after quality filtering. Following resistance status to five antibiotics, samples were classified into 24 sensitive, 24 single resistance, 16 double resistance, 5 triple resistance. Samples were mostly resistant to metronidazole (73.33%; 33/45). Comparation of four groups displayed significantly elevated α-diversity parameters under the multidrug resistance condition (all *P* <0.05). A notable change was observed in triple-resistant compared to sensitive (*P* <0.05) and double-resistant (*P* <0.05) groups. Differences in β-diversity by UniFrac and Jaccard were not significant in terms of the resistance (*P* = 0.113 and *P* = 0.275, respectively). In the triple-resistant group, the relative abundance of *Helicobacter* genera was lower, whereas that of *Streptococcus* increased. Moreover, the linear discriminant analysis effect size (LEfSe) was associated with the presence of *Corynebacterium* and *Saccharimonadales* in the single-resistant group and *Pseudomonas* and *Cloacibacterium* in the triple-resistant group.

**Conclusion:**

Our results suggest that the resistant samples showed a higher trend of diversity and evenness than the sensitive samples. The abundance of *H*. *pylori* in the triple-resistant samples decreased with increasing cohabitation of pathogenic bacteria, which may support antimicrobial resistance. However, antibiotic susceptibility determined by the E-test may not completely represent the resistance status.

## Introduction

Antibiotic resistance has been observed in healthcare-associated and community-acquired infections and has become a global public health challenge. The World Health Organization has acknowledged studies demonstrating a longer duration of hospitalization and increased 30-day mortality in patients with resistant bacteria [1]. The estimated annual cost of this burden is $55 billion every year in United States, namely, $20 and $45 billion in healthcare and loss of productivity, respectively. The cumulative burden could decrease the annual global gross domestic product (GDP) by approximately 1%, with developing countries experiencing losses of 5–7% by 2050 [2]. Moreover, this burden is doubled in the case of multidrug-resistant (MDR) bacteria. MDR strains are often harder and more expensive to handle [3,4]. MDR infections result in 700.000 deaths per year, that may rise to 10 million by 2050 depending on the evolution of resistance patterns and the development of potent antibiotics. The anticipated associated costs amount to 3.8% of the annual GDP (additional 1.2 trillion USD) [5]. Despite global efforts to decrease the overuse of antibiotics, the use of antibiotic classes based on their spectrum of activity is not solely associated with the spread of resistance [6]. Several studies have demonstrated that antibiotics affect the composition and functionality of human microbiota. This is correlated with dysbiosis and the domination of the bacterial composition by pathogenic bacteria, selection of resistant bacteria, and susceptibility to recurrent infections [7]. The relative abundance of gut colonization has been linked to the development of MDR bacteria [8].

*Helicobacter pylori* (*H*. *pylori*) is one of the most common human pathogens, and 50% of the global population is estimated to be infected by it. *H*. *pylori* triggers numerous pathological alterations in the stomach, including peptic ulcer disease, primary gastritis, and gastric cancer [9]. Thus, the eradication of infection can reduce the risk of gastric cancer. The first-line regimen was a triple combination of proton pump inhibitor (PPI) and two antibiotic options: amoxicillin, clarithromycin, metronidazole, and clarithromycin [10]. However, since the late 2000s, *H*. *pylori* eradication rates have decreased because of its increasing resistance to one or more antibiotics [11,12]. A systematic review of Asia-Pacific countries showed that the mean primary *H*. *pylori* resistance rates were 17, 44, 18, 3, and 4% for clarithromycin, metronidazole, levofloxacin, amoxicillin, and tetracycline, respectively [13]. Moreover, triple- and quintuple-MDR *H*. *pylori* strains have been reported in China [14]. These data highlight the worldwide emergence of *H*. *pylori* antibiotic resistance.

The advancement of 16S rRNA sequencing over the years has helped reveal the composition of the gastric microbiota, which helps understand the complex interactions between *H*. *pylori* and other gastric microbiota communities in the gastric microenvironment. Previous studies have linked changes in the gastric microbiome composition between *H*. *pylori*-negative and *H*. *pylori*-positive individuals [15,16]. The existence of the *Helicobacter* genus is associated with an increase in the abundance of other pathogenic populations. Additionally, the gastric microbiota composition is altered during the progression of gastritis to gastric cancer [17,18]. Although antibiotic resistance status is known to affect the gut microbiota [19,20], data related to the gastric environment are still lacking. Therefore, the cohabitation and interplay of the gastric microbiota regarding the antibiotic resistance requires further examination.

Here, we conduct the first microbiome community study on the resistance of *H*. *pylori*-positive to five antibiotics in gastric mucosa samples. *H*. *pylori*-positive biopsy samples with a resistant status were analyzed for diversity and richness. Subsequently, linear discriminant analysis effect size (LEfSe) analysis was conducted to determine the abundance of microbiota.

## Material and methods

### Patients and samples

The *H*. *pylori* infection study was conducted from 2014 to 2016 in Indonesia; a total of 1074 samples were obtained through endoscopy and biopsy sampling combined with our previous results [21]. All subjects were > 18 years old and had dyspeptic symptoms (postprandial fullness, early satiety, epigastric pain, and heartburn) with or without *H*. *pylori* treatment prior to examination. We collected demographic data (age and sex) and a simple diagnosis of dyspepsia using a questionnaire, as described in our previous study [22]. Patients with incomplete specimens were excluded from our study.

We investigated the microbiome on gastric mucosal specimens obtained from the lesser curvature of the antrum, approximately 3 cm from the pyloric ring. The biopsy samples were stored in a transport medium containing 10% glycerol. For the *H*. *pylori* culture, the specimens were homogenized in 500 μL of phosphate-buffered saline, and the remaining homogenized specimens then stored at a temperature of −80°C for DNA extraction later. Histological examination was conducted using biopsy specimens from the corpus and antrum. All the specimens were obtained using Radial Jaw 4 forceps (Boston Scientific).

**Fig 1:**
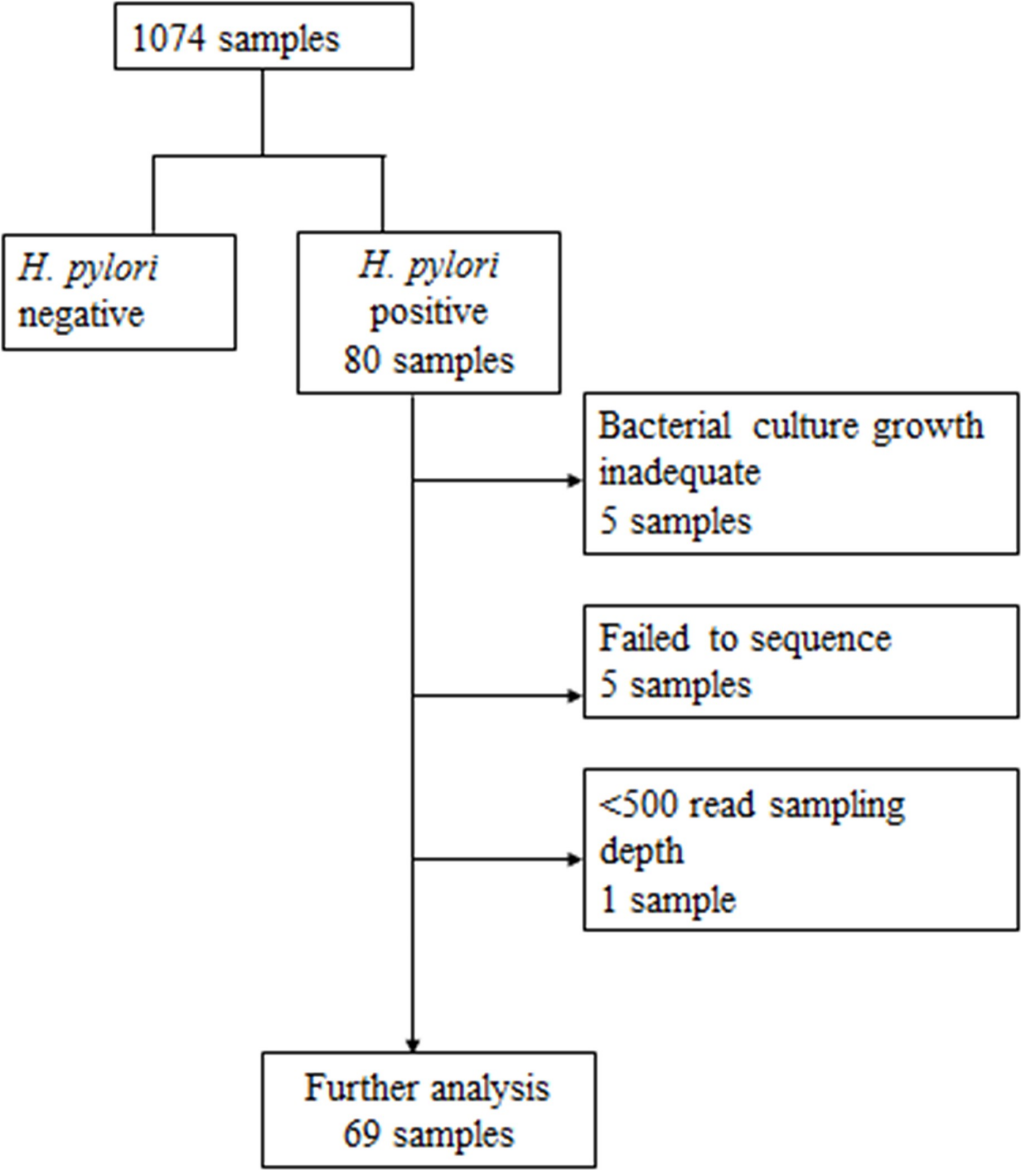
Diagram of the quality filtering of the total biopsy samples combined with the results of our previous study. Of the 1074 samples, 80 samples showed adequate quality and quantity for *H*. *pylori* DNA extraction. Final analysis after determining the sequence quality included 69 samples.

Owing to the low prevalence of *H*. *pylori* infection, we included 80 specimens of *H*. *pylori*-positive from the culture, and the histological analysis involved DNA extraction and sequencing. The final analysis included 69 specimens after quality filtering (Fig 1).

All the participants provided written and verbal consent after signing an informed consent form. The study concept and protocol were approved by the Ethics Committee of the Dr. Soetomo Teaching Hospital (221/Panke. KKE/IX/2012; Surabaya, Indonesia) and the Oita University Faculty of Medicine (Yufu, Japan). All protocols followed the principles of the Declaration of Helsinki involving human subjects.

### Determination of *H*. *pylori* infection

Using cultures of antral biopsy specimens, we determined the infection status based on the *H*. *pylori* culture and histological examination. *H*. *pylori* was isolated from the antral tissues, cultured, and incubated at 37°C for 10 days on a selective agar plate for *H*. *pylori* containing equine serum, soybean peptide, sodium chloride, and selective antibiotics (Nissui Pharmaceutical Co., Japan) in a microaerophilic environment. Colonies were then sub-cultured in antibiotic-free agar medium (Mueller-Hinton II) supplemented with 10% horse blood (Becton Dickinson) in microaerophilic conditions; subsequently, the *H*. *pylori* stock was stored at −80°C in Brucella broth (Difco). All the biopsy specimens were preserved in 10% buffered formalin and embedded in paraffin for histological evaluation. Serial sections were stained with hematoxylin-eosin and May-Giemsa stains, and *H*. *pylori* was detected in samples with bacterial burdens of grade 1 or above.

### Antibiotic susceptibility test

Antibiotic sensitivity measurements were conducted by measuring the minimum inhibitory concentration (MIC) of five antibiotics (amoxicillin, levofloxacin, metronidazole, clarithromycin, and tetracycline), as described in our previous study [23]. Procedural tests were conducted using an E-test (Biomerieux, France). First, the selected *H*. *pylori* cultures were sub-cultured thrice on a Brucella agar plate supplemented with 7% horse blood without antibiotics under microaerophilic conditions overnight. Subsequently, *H*. *pylori* colonies were collected in phosphate-buffered saline, customized to a 3.0 McFarland concentration, and embedded on 5% horse blood-supplemented Mueller Hinton agar. E-test strips containing five types of antibiotics were patched in the center. The antibiotic concentrations were as follows: 0.016–256 mg/L for clarithromycin, amoxicillin, tetracycline, and metronidazole, respectively, and 0.002–32 mg/L for levofloxacin. The area of inhibition was measured after 72 h. All sensitivities were determined according to the clinical breakpoint of the European Committee on Antimicrobial Susceptibility Testing (EUCAST) [24].

### DNA extraction and PCR amplification of 16S rRNA sequences

DNA extraction and PCR amplification were conducted as previously described [25]. DNA was extracted using the QIAGEN DNeasy Blood & Tissue Kit (QIAGEN) and concentrated using the DNA Clean & Concentrator (QIAGEN) (Zymo Research). The DNA was extracted and maintained at a temperature of 20°C. The 16S rRNA gene library was prepared according to the manufacturer’s instructions (Illumina, San Diego, CA, USA). Universal primer 341F was employed to amplify the V3-V4 regions of the bacterial 16S rRNA gene, and 17 polymerase chain reaction (PCR) amplifications were conducted using KAPA HiFi HotStart Ready Mix (KAPA Biosystem Inc. Eight PCR cycles were conducted using the Nextera XT Index kit (Illumina Inc.), and Agencourt AMPure XP magnetic beads (Beckman Coulter) were used for amplicon purification. The DNA library was validated using the MCE-202 MultiNA system (Shimadzu) and QuantiFluor dsDNA system (Promega Corporation). The pooled 5 pM DNA library was denatured with 0.2 N of NaOH and combined with PhiX Control v3 (Illumina Inc.) to a final concentration of 15%, according to the protocol of Illumina. Paired-end sequencing was conducted using the MiSeq platform (Illumina Inc.) and MiSeq Reagent Kit version 3 (2300 bp paired-end reads; Illumina Inc.).

### Sequence data analysis

Reads obtained from the Illumina MiSeq platform were analyzed using the QIIME2 (Version 2021.2) pipeline [26]. The demultiplexed reads were uploaded, trimmed, and filtered using Cutadapt to remove low-quality reads. The Deblur pipeline integrated into QIIME2 was used for denoising, chimera removal, and clustering. The output was classified for taxonomy and allocated to the SILVA 138 reference database with 99% identity [27]. These sequences were aligned using MAFFT, and a phylogenetic tree was constructed using FastTree [28,29]. We imported the operational taxonomic unit (OTU) table, representative sequences, and phylogenetic tree as artifacts into the QIIME2 analysis platform (https://qiime2.org). The α-diversity including the observed richness, Simpson and Shannon diversity indexes, and Pielou’s evenness were analyzed using the alpha significance QIIME diversity. The Wilcoxon signed-rank test was used to compare the diversity indexes. The plot was drawn in the “ggplot2” package of the R environment (ver 4.02).

The OTU tables, metadata, taxonomy, and phylogenetic tree were exported into the phyloseq object using biom-convert and “phyloseq” package of the R environment for beta diversity and abundance analysis. The β-diversity analysis measured the unweighted UniFrac and Bray-Curtis distances. Principal component analysis was conducted using the R software. The Adonis function from the “vegan” package was used to conduct permutational analysis of variance (PERMANOVA) with the Bonferroni correction. Using the “phyloseqCompanion” package in R, we conducted LEfSe to determine OTUs that are likely to explain differences between sensitive, single-, double-, and triple-resistant strains. We also used the Kruskal-Wallis test to determine the presence of any differences in the relative abundances of specific OTUs in the resistant strains.

## Results

### Subjects and antibiotic resistance status

Among the 69 subjects who were chosen for further analysis, 30 (43.47%) and 39 (56.52%) were male and female, respectively. Most patients were diagnosed with gastritis (n = 58, 84.05%) and had no history of *H*. *pylori* eradication (n = 48, 69.56%) (Table 1). The drug profiles were based on the E-test of antibiotic susceptibility to five antibiotics: amoxicillin, levofloxacin, metronidazole, clarithromycin, and tetracycline. We found that strains were most resistant to metronidazole (33/45, 73.33%) and least resistant to amoxicillin and tetracycline (4/45, 8.88% and 2/49, 4.44%, respectively). Based on the resistance to five types of antibiotics, 24, 24, 16, and 5 sample cultures were classified as sensitive, single-resistant, double-resistant, and triple-resistant groups.

**Table 1:** Subject characteristics.

Parameter	n = 69 (%)
**Gender**	
Male	30 (43.47%)
Female	39 (56.52%)
**Age (mean±SD)**	52.23 ± 12.64
**Eradication history**	
Yes	13 (18.84%)
No	48 (69.56%)
Unknown	8 (11.59%)
**Diagnosis**	
GERD	2 (2.89%)
Gastritis	58 (84.05%)
Duodenitis	1 (1.44%)
Gastric ulcer	7 (10.14%)
Gastric cancer	1 (1.44%)
**Resistance Status**	
Sensitive	24 (34.78%)
**Resistance Status**	
Single	24 (34.78%)
Double	16 (23.18%)
Triple	5 (7.24%)
**Antibiotic**	n = 45 (%)
Amoxicillin	4/45
Clarithromycin	16/45
Metronidazole	33/45
Tetracycline	2/45
Levofloxacin	14/45

### Alpha diversity of the gastric microbiome in susceptible and resistant samples

**Fig 2:**
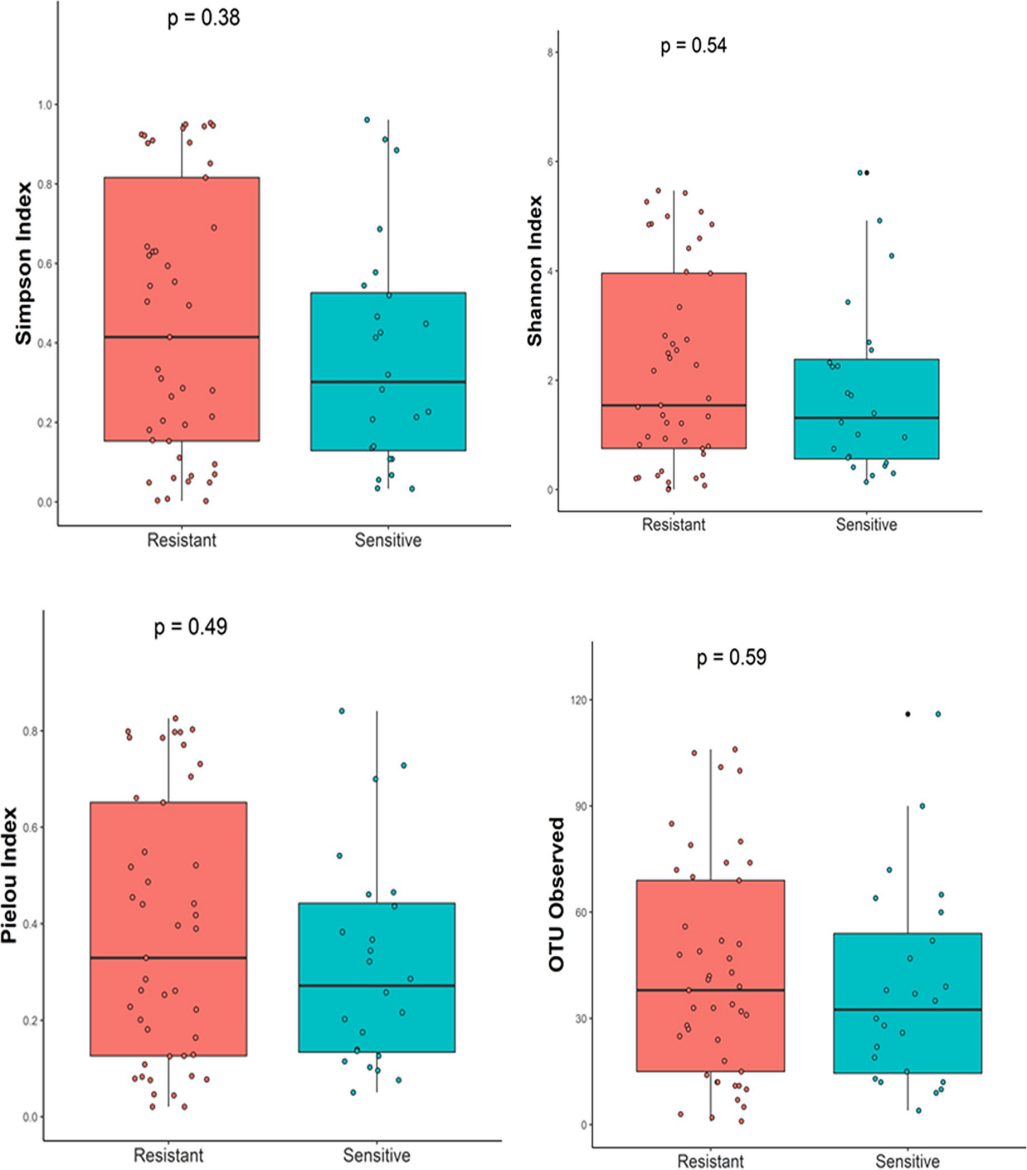
Plots displaying the alpha diversity parameters of gastric microbiota between sensitive and resistant *H*. *pylori* individuals measured by Shannon and Simpson indexes; the evenness and richness were measured by the Pielou’s index and observed OTUs. The boxes represent the interquartile range (IQR) between the first and third quartiles (25th and 75th percentiles, respectively). The vertical line inside the box denotes the median.

The analyzed data were set at a minimum sampling depth of 500 reads for diversity normalization, and no samples were excluded. To investigate the general condition, we analyzed the gastric microbiota community according to the susceptibility status. Overall, we observed a higher tendency for diversity and evenness, but this was not statistically significant in the resistant group compared with the sensitive group. Fig 2 shows the Shannon diversity index (*P* = 0.54), Simpson index (*P* = 0.38), and Pielou’s evenness index (*P* = 0.3664). Bacterial community richness at the OTU level was also comparable between the groups (*P* = 0.5119).

### Gastric microbiota diversity among resistant groups

**Fig 3:**
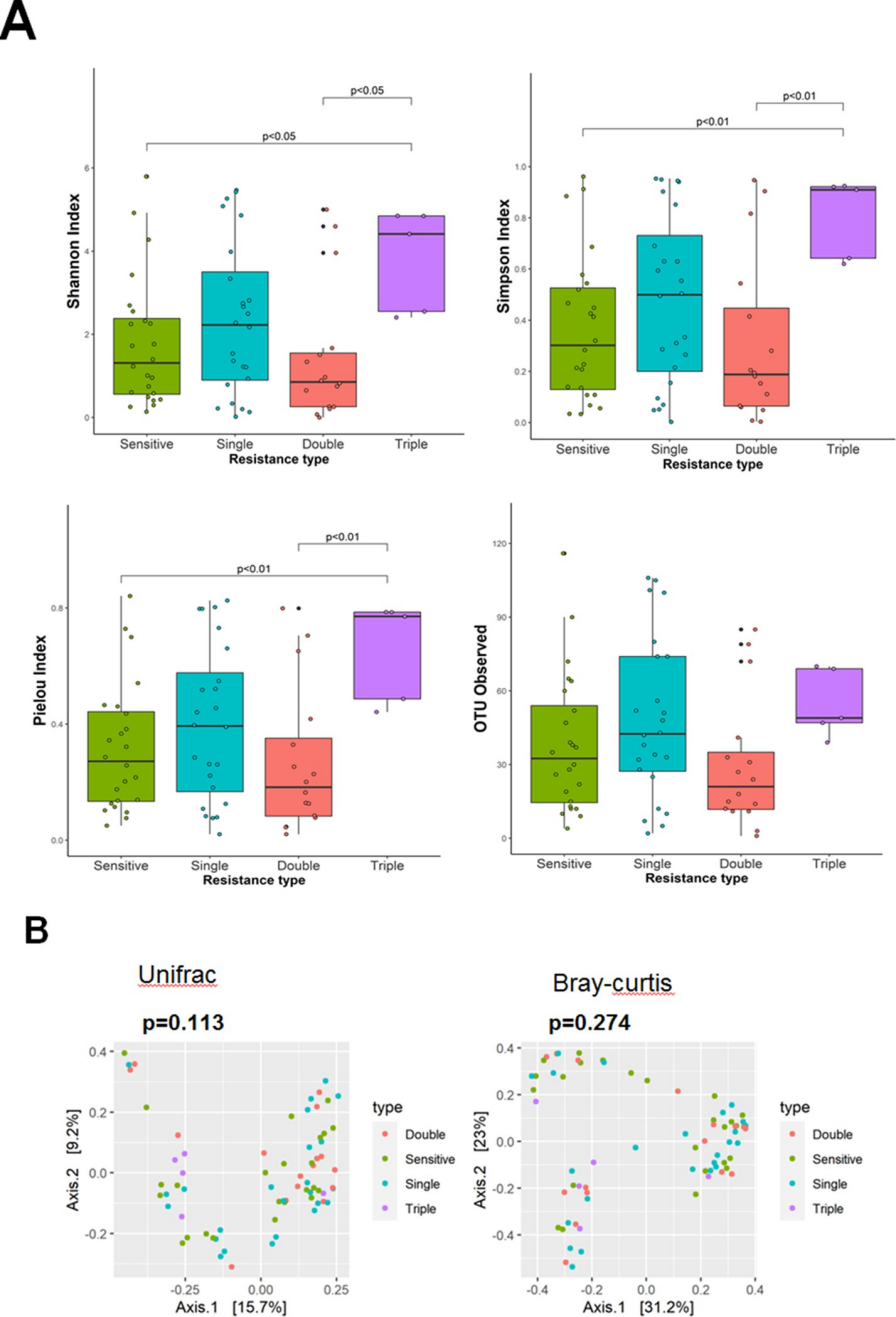
Gastric microbiome diversity of different resistance patterns. (A). Boxplot displaying the alpha diversity measured by the Shannon index, Simpson index, and Pielou’s index, and observed OTUs. It showed significant differences in the diversity measured by the Shannon and Simpson indexes and the community evenness measured by Pielou’s index. (B) Beta diversity measured by UniFrac and Bray-Curtis distances indicated that the gastric community cannot be separated into particular clusters.

We further investigated the diversity of drug resistance to the five antibiotics using the E-test. Our analysis indicated significant differences in the alpha diversity of the Shannon (*P* = 0.04) and Simpson (*P* = 0.032) indexes, and evenness of Pielou’s index (*P* = 0.025) among the groups. The pair-wise comparison of sensitive and triple-resistant strains showed an increasing trend of diversity richness for MDR strains, and double- and triple-resistant strains showed significant results in the Shannon index (Fig 3A). A similar tendency was observed for the Simpson index, which was concordant with the Shannon index. The pair-wise comparison of the evenness between sensitive and triple-resistant groups (*P* <0.01) and the comparison between double- and triple-resistant groups (*P* <0.01) were also conducted. The α-diversities showed that triple-MDR strains had the highest diversity and evenness trends. In contrast, double-resistant group displayed a lower trend in the diversity and evenness compared with the sensitive and other resistant groups. Generally, the observed OTUs were comparable between groups (Fig 3A).

We evaluated differences in the β-diversity analysis distance plot. Both UniFrac and Bray-Curtis distances revealed that the clusters appeared separate but were not significantly correlated with drug resistance (*P* = 0.113 and *P* = 0.274, respectively), as indicated by PERMANOVA (Fig 3B).

### Relative abundances of gastric microbiota are affected in the presence of resistant *H*. *pylori*

**Fig 4:**
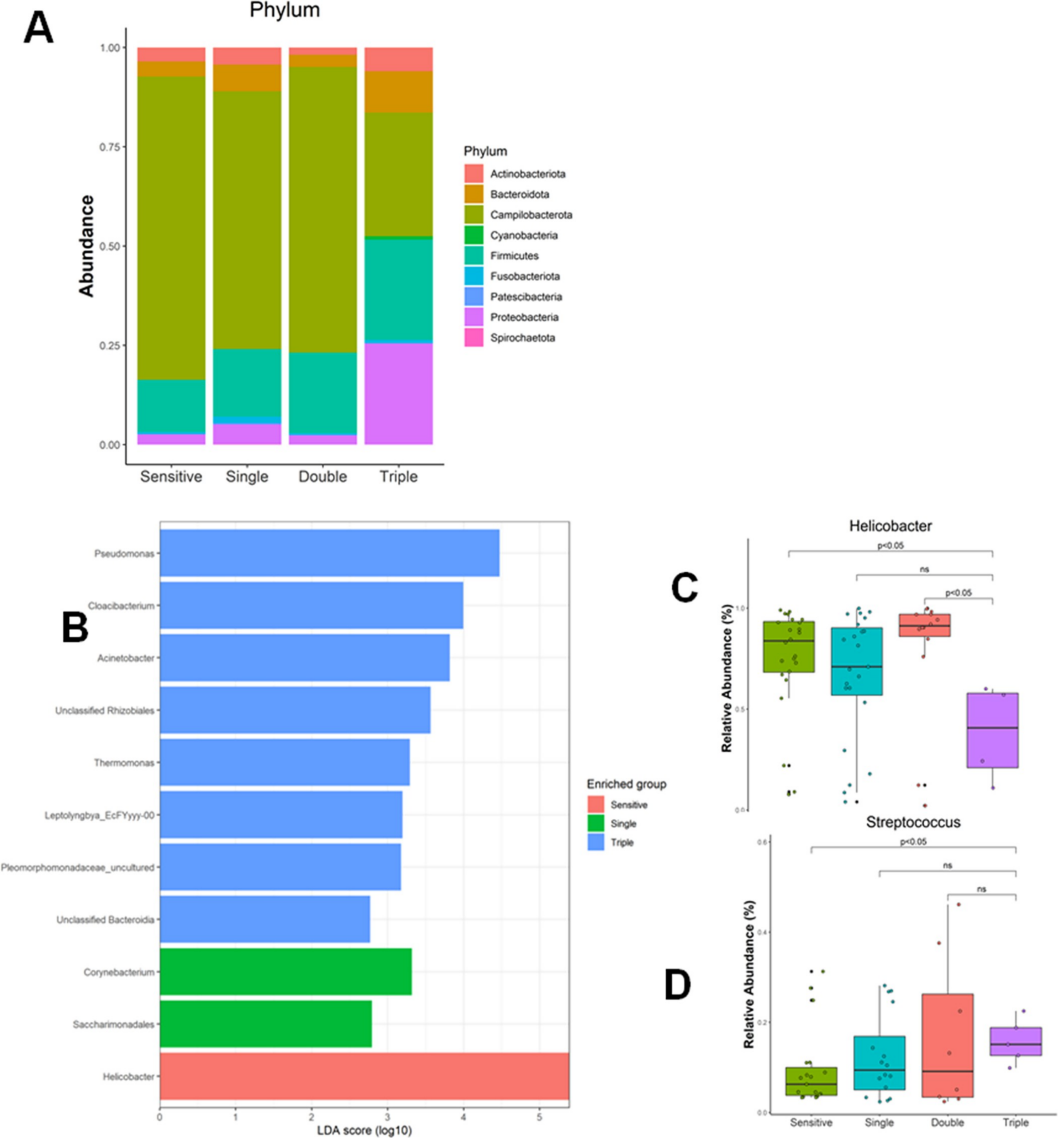
Characteristics of the microbial composition between resistance types. (A) Taxa comparison in terms of the phylum and family. A lower proportion of Campylobacteria was observed with an increase in Proteobacteria from single resistance to triple resistance. (B) Analysis of LDA by LEFse at the genus level found possible biomarkers in resistance strains. Enrichment of Pseudomonas and Cloacibacterium was observed in triple resistance and that of Corynebacterium was observed in single resistance. (C & D) Boxplots illustrating the abundance of two major genera across resistance groups. Depleted abundance of *Helicobacter* was observed in the triple resistance group.

As all samples were *H*. *pylori*-positive, the comparison of relative abundance indicated that the most abundant phylum in all groups was Campylobacterota. The major human stomach microbiota communities, Actinobacteria, Bacteroidetes, and Firmicutes [30] were observed across the groups. However, an increase in the proportion of Proteobacteria was observed in the triple-resistant group (Fig 4A).

Linear discriminant analysis (LDA) at the genus level was conducted for taxa with an abundance of at least 2% of the population. Several taxa characterizing each disease group were observed with an LDA score cutoff of 5.0 (Fig 4B). In addition to *Helicobacter*, single resistance was enriched in *Corynebacterium* and *Saccharimonadales*, whereas triple resistance was highly enriched in *Pseudomonas* and *Cloacibacterium*. Additionally, at the genus level, the triple-resistant group had a significantly depleted relative abundance of *Helicobacter* compared with the other groups and higher *Streptococcus* compared with the sensitive group (Fig 4C and 4D).

## Discussion

The stomach was thought to be sterilized by commensal bacteria because of the low pH of gastric acid. The discovery of *H*. *pylori* broke the paradigm and subsequently changed perspectives regarding the microbiota community in the gastric niche [31]. *H*. *pylori* is a class I carcinogen associated with gastric cancer. This infection alters the physiological conditions of the gastric environment to serve other bacterial habitats [9]. As the contribution of microbiota to human diseases is increasing, it is important to analyze distinct differences in the gastric microbiome diversity, especially in terms of the *H*. *pylori* resistance status.

To the best of our knowledge, microbiome analysis of MDR strains remains limited, and this exploration is the first of its kind for gastric. This study highlights a few findings related to the resistance status. Overall, the resistant strains showed a higher trend in diversity and community evenness compared to the sensitive group. Subsequently, based on the resistance type, there was an increase in the α-diversity index among the MDR. The diversity and richness significantly increased in triple-resistant strains compared with sensitive and single-resistant strains (Fig 3). Notably, our results showed that double-resistant strains had the lowest diversity index. Further research is required to determine the effects of resistance on microbial communities. However, the cluster distance in the β-diversity index was not correlated with the resistance status. Our results indicate that dominant bacteria could suppress the commensal microbiota and increase the number of pathogenic bacteria.

The virulence factors CagA and VacA can alter the gastric microbiota and immune phenotypes previously attributed to *H*. *pylori* infection in the stomach [32]. Moreover, previous studies have demonstrated that resistance-carrying microbiomes (resistomes) had higher diversity and relative abundance trends. The group found that resistome-carrying healthy outpatient children had more *Enterobacteriaceae* spp. with extended-spectrum beta-lactamase (ESBL) gene potential [19].

In the recent study, patients were mostly diagnosed with gastritis with no prior eradication history. We could not specify deeper type of any previous dyspeptic therapy nor antibiotic treatment of patients. Antibiotic activity plays a role in the microbiota composition. Strains showed major antibiotic resistant to metronidazole, followed by clarithromycin and levofloxacin in the E-test. Clinically, metronidazole has been extensively used to target anaerobic bacteria, such as *Bacteroidetes* spp. [33], whereas clarithromycin targets anaerobic and extensive gram-positive bacteria [34]. Investigating *H*. *pylori* resistance to treatment in Indonesia revealed a high distribution of metronidazole and levofloxacin resistance. The 23S rRNA A2143G mutation for metronidazole is associated with clarithromycin resistance [35]. This may also combine with PPIs influence towards lower gastric acidity and development of dysbiosis [36]. The consequences and mechanism of any previous therapy of a patient before biopsy were unclear, but those could affect the bacterial culture and susceptibility, resulting in the deviation of the double-resistant out of pattern.

The abundance of phyla in all groups showed that the core microbiome phyla found in healthy individuals (Firmicutes and Bacteroidetes) were suppressed by Campylobacterota dominance, as shown in Fig 4. Most studies agree that *H*. *pylori* infection occupies most of the gastric niche [15,37]. Notably, triple resistance was associated with an increased proportion of Proteobacteria, including many common gram-negative pathogens. LDA indicated that *Pseudomonas* was the most differentially abundant genus in triple-resistant group. A higher abundance of *Streptococcus* was also observed. There is a possibility of subjects that possessed MDR *H*. *pylori* could also be infected with other MDR bacteria and their augmented abundance repressed the growth of *H*. *pylori*, as shown in our results. A previous study reported that the abundance of gram-negative bacteria, such as *Escherichia coli*, *Enterobacter cloacae*, and *Salmonella enterica* increased in patients with MDR bacterial infections [20]. The dominance of *Streptococcus* and various Proteobacteria is a marker of persistent dysbiosis, which subsequently increases bacteremia [38,39]. Those gram-negative bacteria abundance may be related to the transfer of antibiotic resistance [40], wherein gene variants may be present in *H*. *pylori* [41]. This is supported by the ability of *H*. *pylori* to form biofilms, which are known to reduce susceptibility compared with the planktonic phase [42]. Moreover, a possible association may exist between biofilm formation and interspecies gene transfer [43].

This study had several limitations. First, the findings presented here were analyzed using 16S rRNA sequencing; thus, we could not determine the presence of drug resistance in other organisms. However, this can serve as a preliminary view of the gastric microbial community in patients with MDR strains. Further investigation using the shotgun metagenome is necessary to confirm the presence of antimicrobial resistance genes possessed by all bacteria present in the environment and help better understand the reciprocal effect. Second, our previous prospective population-based study demonstrated the low prevalence of *H*. *pylori* in Indonesia [22,44]. Moreover, this was a cross-sectional study where we found low triple-resistant samples (n = 5), and most resistances were toward metronidazole; hence, pre- and post-antibiotic use cohorts are required to provide a more objective perspective.

In conclusion, recent data indicate that dysbiosis due to multidrug use and cohabitation compositions may be correlated. The resistance status of *H*. *pylori* was correlated with the enriched diversity of the gastric microbiome composition, where the abundance of non-*H*. *pylori* pathogens increased, especially in triple-resistant strains. Given this possibility, clinicians should be mindful of antibiotic combination treatments because drug resistance in the gut environment could affect the gastrointestinal niche and probably induce drug resistance in other bacteria.

## References

[1] World Health Organization. Antimicrobial resistance: global report on surveillance. 2014; 256.

[2] Dadgostar P. Antimicrobial Resistance: Implications and Costs. Infection and drug resistance. 2019;12: 3903–3910. 10.2147/IDR.S234610 PMC6929930 31908502

[3] Lim C, et al. Epidemiology and burden of multidrug-resistant bacterial infection in a developing country. eLife. 2016 Sept;5. doi: 10.7554/eLife.18082 27599374 PMC5030096

[4] Stewardson AJ, et al. Effect of carbapenem resistance on outcomes of bloodstream infection caused by Enterobacteriaceae in low-income and middle-income countries (PANORAMA): a multinational prospective cohort study. The Lancet Infectious Diseases. 2019;19(6): 601–610. doi: 10.1016/S1473-3099(18)30792-8 31047852

[5] Serra-Burriel M, et al. Impact of multi-drug resistant bacteria on economic and clinical outcomes of healthcare-associated infections in adults: Systematic review and meta- analysis PLoS One. 2020;15(1): E0227139. doi: 10.1371/journal.pone.0227139 31923281 PMC6953842

[6] Klein EY, et al. Global increase and geographic convergence in antibiotic consumption between 2000 and 2015. Proceedings of the National Academy of Sciences of the United States of America. 2018;115(15): E3463–E3470. doi: 10.1073/pnas.1717295115 29581252 PMC5899442

[7] Pilmis B, Le Monnier A, Zahar JR. Gut Microbiota, Antibiotic Therapy and Antimicrobial Resistance: A Narrative Review. Microorganisms. 2020;8(2). doi: 10.3390/microorganisms8020269 32079318 PMC7074698

[8] Ruppe E, et al. Relative fecal abundance of extended-spectrum-beta-lactamase-producing Escherichia coli strains and their occurrence in urinary tract infections in women. Antimicrob Agents Chemother. 2013;57(9): 4512–7. 23836184 10.1128/AAC.00238-13 PMC3754361

[9] Wong U, McLean LP. Diagnosis and Management of Helicobacter pylor. J Clin Gastroenterol Treat. 2016;2(15).

[10] Hunt RH, et al. Helicobacter pylori in developing countries. World Gastroenterology Organisation Global Guideline. J Gastrointestin Liver Dis. 2011;20(3): 299–304. 21961099

[11] Matsumoto H, Shiotani A, Graham DY. Current and Future Treatment of Helicobacter pylori Infections. Advances in experimental medicine and biology. 2019;1149: 211–225. doi: 10.1007/5584_2019_367 31016626 PMC6918954

[12] Savoldi A, et al. Prevalence of Antibiotic Resistance in Helicobacter pylori: A Systematic Review and Meta-analysis in World Health Organization Regions. Gastroenterology. 2018;155(5): 1372–1382.e17. 29990487 10.1053/j.gastro.2018.07.007 PMC6905086

[13] Kuo YT, et al. Primary antibiotic resistance in Helicobacter pylori in the Asia-Pacific region: a systematic review and meta-analysis. Lancet Gastroenterol Hepatol. 2017;2(10): 707–715. doi: 10.1016/S2468-1253(17)30219-4 28781119

[14] Wang D, et al. The antibiotic resistance of Helicobacter pylori to five antibiotics and influencing factors in an area of China with a high risk of gastric cancer. BMC microbiology. 2019; 19(1): 152–152. doi: 10.1186/s12866-019-1517-4 31272365 PMC6611032

[15] Maldonado-Contreras A, et al. Structure of the human gastric bacterial community in relation to Helicobacter pylori status. The ISME journal. 2011;5(4): 574–579. doi: 10.1038/ismej.2010.149 20927139 PMC3105737

[16] Zhao Y, et al. Helicobacter pylori infection alters gastric and tongue coating microbial communities. Helicobacter. 2019;24(2): e12567–e12567. doi: 10.1111/hel.12567 30734438 PMC6593728

[17] Yang J, et al. Role of the Gastric Microbiome in Gastric Cancer: From Carcinogenesis to Treatment. Frontiers in Microbiology. 2021;12. doi: 10.3389/fmicb.2021.641322 33790881 PMC8005548

[18] Zhang X, et al. Alterations of Gastric Microbiota in Gastric Cancer and Precancerous Stages. Front Cell Infect Microbiol. 2021;11: 559148. doi: 10.3389/fcimb.2021.559148 33747975 PMC7966516

[19] Andersen H, et al. Use of Shotgun Metagenome Sequencing To Detect Fecal Colonization with Multidrug-Resistant Bacteria in Children. J Clin Microbiol. 2016;54(7): 1804–1813. doi: 10.1128/JCM.02638-15 27122381 PMC4922122

[20] Afridi OK, Ali J, Chang JH. Resistome and microbial profiling of pediatric patient’s gut infected with multidrug-resistant diarrhoeagenic Enterobacteriaceae using next-generation sequencing; the first study from Pakistan. Libyan J Med. 2021;16(1): 1915615. doi: 10.1080/19932820.2021.1915615 33877031 PMC8078919

[21] Miftahussurur M, et al. Gastric microbiota and Helicobacter pylori in Indonesian population. Helicobacter. 2020;25(4): e12695. doi: 10.1111/hel.12695 32395907

[22] Syam AF, et al. Risk Factors and Prevalence of Helicobacter pylori in Five Largest Islands of Indonesia: A Preliminary Study. PLoS One. 2015;10(11): e0140186. doi: 10.1371/journal.pone.0140186 26599790 PMC4658100

[23] Fauzia KA, et al. Biofilm Formation and Antibiotic Resistance Phenotype of Helicobacter pylori Clinical Isolates. Toxins (Basel). 2020;12(8). doi: 10.3390/toxins12080473 32722296 PMC7472329

[24] EUCAST, EUCAS clinical brakpoints for Helicobacter pylori. European Society of Clinical Microbiology and Infectious Diseases. 2011.

[25] Miftahussurur M, et al. Gastric microbiota and Helicobacter pylori in Indonesian population. Helicobacter. 2020;25(4): e12695. doi: 10.1111/hel.12695 32395907

[26] Bolyen E, et al. Reproducible, interactive, scalable and extensible microbiome data science using QIIME 2. Nat Biotechnol. 2019;37(8): 852–857. doi: 10.1038/s41587-019-0209-9 31341288 PMC7015180

[27] Yilmaz P, et al. The SILVA and “all-species living tree project (LTP)” taxonomic frameworks. Nucleic acids research. 2014;42(D1): D643–D648. doi: 10.1093/nar/gkt1209 24293649 PMC3965112

[28] Katoh K, et al. MAFFT version 5: improvement in accuracy of multiple sequence alignment. Nucleic acids research. 2005;33(2): 511–518. doi: 10.1093/nar/gki198 15661851 PMC548345

[29] Price MN, Dehal PS, Arkin AP. FastTree 2–approximately maximum-likelihood trees for large alignments. PloS one. 2010;5(3): e9490. doi: 10.1371/journal.pone.0009490 20224823 PMC2835736

[30] Tap J, et al. Towards the human intestinal microbiota phylogenetic core. Environ Microbiol. 2009;11(10): 2574–84. doi: 10.1111/j.1462-2920.2009.01982.x 19601958

[31] Yang I, Nell S, Suerbaum S. Survival in hostile territory: the microbiota of the stomach. FEMS Microbiol Rev. 2013;37(5): 736–61. doi: 10.1111/1574-6976.12027 23790154

[32] Jones TA, et al. The bacterial virulence factor CagA induces microbial dysbiosis that contributes to excessive epithelial cell proliferation in the Drosophila gut. PLoS Pathog. 2017;13(10): e1006631. doi: 10.1371/journal.ppat.1006631 29049360 PMC5648253

[33] Hsu PI, et al. Helicobacter pylori eradication with bismuth quadruple therapy leads to dysbiosis of gut microbiota with an increased relative abundance of Proteobacteria and decreased relative abundances of Bacteroidetes and Actinobacteria. Helicobacter. 2018;23(4): e12498. doi: 10.1111/hel.12498 29897654

[34] Elvers KT, et al. Antibiotic-induced changes in the human gut microbiota for the most commonly prescribed antibiotics in primary care in the UK: a systematic review. BMJ Open. 2020;10(9): e035677. doi: 10.1136/bmjopen-2019-035677 32958481 PMC7507860

[35] Miftahussurur M, et al. Surveillance of Helicobacter pylori Antibiotic Susceptibility in Indonesia: Different Resistance Types among Regions and with Novel Genetic Mutations. PLoS ONE. 2016;11(12): e0166199. doi: 10.1371/journal.pone.0166199 27906990 PMC5131997

[36] Gomez-Ramirez U, et al. Role of Helicobacter pylori and Other Environmental Factors in the Development of Gastric Dysbiosis. Pathogens. 2021;10(9): 1203. doi: 10.3390/pathogens10091203 34578235 PMC8467233

[37] Andersson AF, et al. Comparative analysis of human gut microbiota by barcoded pyrosequencing. PLoS One. 2008;3(7): e2836. doi: 10.1371/journal.pone.0002836 18665274 PMC2475661

[38] Taur Y, et al. Intestinal domination and the risk of bacteremia in patients undergoing allogeneic hematopoietic stem cell transplantation. Clin Infect Dis. 2012;55(7): 905–14. doi: 10.1093/cid/cis580 22718773 PMC3657523

[39] Annavajhala MK, et al. Colonizing multidrug-resistant bacteria and the longitudinal evolution of the intestinal microbiome after liver transplantation. Nat Commun. 2019;10(1): 4715. doi: 10.1038/s41467-019-12633-4 31624266 PMC6797753

[40] Wang Y, et al. Gene sharing among plasmids and chromosomes reveals barriers for antibiotic resistance gene transfer. Philosophical Transactions of the Royal Society B: Biological Sciences. 2022;377(1842): 20200467. 34839702 10.1098/rstb.2020.0467 PMC8628082

[41] Mannion A, et al. Helicobacter pylori Antimicrobial Resistance and Gene Variants in High- and Low-Gastric-Cancer-Risk Populations. Journal of Clinical Microbiology. 2021;59(5): e03203–20. doi: 10.1128/JCM.03203-20 33692136 PMC8091839

[42] Yonezawa H, et al. Impact of Helicobacter pylori biofilm formation on clarithromycin susceptibility and generation of resistance mutations. PLoS One. 2013;8(9): e73301. doi: 10.1371/journal.pone.0073301 24039906 PMC3765302

[43] Lee KWK, et al. Biofilm development and enhanced stress resistance of a model, mixed-species community biofilm. The ISME Journal. 2014;8(4): 894–907. doi: 10.1038/ismej.2013.194 24152718 PMC3960537

[44] Miftahussurur M, et al. Gastric mucosal status in populations with a low prevalence of Helicobacter pylori in Indonesia. PLoS One. 2017;12(5): e0176203. doi: 10.1371/journal.pone.0176203 28463979 PMC5413002

